# Supplementary material for: A Critical View of the Application of the APEX Software (Aqueous Photochemistry of Environmentally-Occurring Xenobiotics) to Predict Photoreaction Kinetics in Surface Freshwaters
Source: Molecules. 2019 Dec 18;25(1):9. doi: 10.3390/molecules25010009 (PMC7017383; doi:10.3390/molecules25010009)
Supplement: Supplementary file 1 [file molecules-25-00009-s001.zip › 3.2.4_gcc-4.4.0.zip!/3.2.4_gcc-4.4.0/3.2.4_gcc-4.4.0/doc/octave/html/liboctave/Constructors-and-Assignment.html]

Constructors and Assignment - Octave C++ Classes


Previous: Arrays,
Up: Arrays


---

### 3.1 Constructors and Assignment

— Constructor: **Array<T>** (void)  
> Create an array with no elements.

— Constructor: **Array<T>** (int n [, const T &val])  
> Create an array with n elements. If the optional argument
> val is supplied, the elements are initialized to val;
> otherwise, they are left uninitialized. If n is less than zero,
> the current error handler is invoked (see Error Handling).

— Constructor: **Array<T>** (const Array<T> &a)  
> Create a copy of the Array<T> object a. Memory for the
> Array<T> class is managed using a reference counting scheme, so
> the cost of this operation is independent of the size of the array.

— Assignment on Array<T>: Array<T>& **operator =** (const Array<T> &a)  
> Assignment operator. Memory for the Array<T> class is managed
> using a reference counting scheme, so the cost of this operation is
> independent of the size of the array.

— Method on Array<T>: int **capacity** (void) const  
— Method on Array<T>: int **length** (void) const  
> Return the length of the array.

— Method on Array<T>: T& **elem** (int n)  
— Method on Array<T>: T& **checkelem** (int n)  
> If n is within the bounds of the array, return a reference to the
> element indexed by n; otherwise, the current error handler is
> invoked (see Error Handling).

— Indexing on Array<T>: T& **operator ()** (int n)

— Method on Array<T>: T **elem** (int n) const  
— Method on Array<T>: T **checkelem** (int n) const  
> If n is within the bounds of the array, return the value indexed
> by n; otherwise, call the current error handler.
> See Error Handling.

— Indexing on Array<T>: T **operator ()** (int n) const

— Method on Array<T>: T& **xelem** (int n)  
— Method on Array<T>: T **xelem** (int n) const  
> Return a reference to, or the value of, the element indexed by n.
> These methods never perform bounds checking.

— Method on Array<T>: void **resize** (  
> Change the size of the array to be n elements. All elements are
> unchanged, except that if n is greater than the current size and
> the optional argument val is provided, the additional elements are
> initialized to val; otherwise, any additional elements are left
> uninitialized. In the current implementation, if n is less than
> the current size, the length is updated but no memory is released.

— Method on Array<T>: const T\* **data** (void) const


— Constructor: **Array2<T>** Array2<T> Array2 (void)  
— Constructor: **Array2<T>** (int n, int m)  
— Constructor: **Array2<T>** (int n, int m, const T &val)  
— Constructor: **Array2<T>** (const Array2<T> &a)  
— Constructor: **Array2<T>** (const DiagArray<T> &a)

— Assignment on Array2<T>: Array2<T>& **operator =** (const Array2<T> &a)

— Method on Array2<T>: int **dim1** (void) const  
— Method on Array2<T>: int **rows** (void) const

— Method on Array2<T>: int **dim2** (void) const  
— Method on Array2<T>: int **cols** (void) const  
— Method on Array2<T>: int **columns** (void) const

— Method on Array2<T>: T& **elem** (int i, int j)  
— Method on Array2<T>: T& **checkelem** (int i, int j)

— Indexing on Array2<T>: T& **operator ()** (int i, int j)


— Method on Array2<T>: void **resize** (int n, int m)  
— Method on Array2<T>: void **resize** (int n, int m, const T &val)

— Constructor: **Array3<T>** (void)  
— Constructor: **Array3<T>** (int n, int m, int k)  
— Constructor: **Array3<T>** (int n, int m, int k, const T &val)  
— Constructor: **Array3<T>** (const Array3<T> &a)

— Assignment on Array3<T>: Array3<T>& **operator =** (const Array3<T> &a)

— Method on Array3<T>: int **dim1** (void) const  
— Method on Array3<T>: int **dim2** (void) const  
— Method on Array3<T>: int **dim3** (void) const

— Method on Array3<T>: T& **elem** (int i, int j, int k)  
— Method on Array3<T>: T& **checkelem** (int i, int j, int k)

— Indexing on Array3<T>: T& **operator ()** (int i, int j, int k)


— Method on Array3<T>: void **resize** (int n, int m, int k)  
— Method on Array3<T>: void **resize** (int n, int m, int k, const T &val)

— Constructor: **DiagArray<T>** (void)  
— Constructor: **DiagArray<T>** (int n)  
— Constructor: **DiagArray<T>** (int n, const T &val)  
— Constructor: **DiagArray<T>** (int r, int c)  
— Constructor: **DiagArray<T>** (int r, int c, const T &val)  
— Constructor: **DiagArray<T>** (const Array<T> &a)  
— Constructor: **DiagArray<T>** (const DiagArray<T> &a)

— Assginment on DiagArray<T>&: **operator =** (const DiagArray<T> &a)

— Method on DiagArray<T>: int **dim1** (void) const  
— Method on DiagArray<T>: int **rows** (void) const

— Method on DiagArray<T>: int **dim2** (void) const  
— Method on DiagArray<T>: int **cols** (void) const  
— Method on DiagArray<T>: int **columns** (void) const

— Method on DiagArray<T>: T& **elem** (int r, int c)  
— Method on DiagArray<T>: T& **checkelem** (int r, int c)

— Indexing on DiagArray<T>: T& **operator ()** (int r, int c)


— Method on DiagArray<T>: void **resize** (int n, int m)  
— Method on DiagArray<T>: void **resize** (int n, int m, const T &val)


The real and complex `ColumnVector` and `RowVector` classes
all have the following functions. These will eventually be part of an
`MArray<T>` class, derived from the `Array<T>` class. Then
the `ColumnVector` and `RowVector` classes will be derived
from the `MArray<T>` class.

Element by element vector by scalar ops.

— : RowVector **operator +** (const RowVector &a, const double &s)  
— : RowVector **operator -** (const RowVector &a, const double &s)  
— : RowVector **operator \*** (const RowVector &a, const double &s)  
— : RowVector **operator /** (const RowVector &a, const double &s)

Element by element scalar by vector ops.

— : RowVector **operator +** (const double &s, const RowVector &a)  
— : RowVector **operator -** (const double &s, const RowVector &a)  
— : RowVector **operator \*** (const double &s, const RowVector &a)  
— : RowVector **operator /** (const double &s, const RowVector &a)

Element by element vector by vector ops.

— : RowVector **operator +** (const RowVector &a, const RowVector &b)  
— : RowVector **operator -** (const RowVector &a, const RowVector &b)

— : RowVector **product** (const RowVector &a, const RowVector &b)  
— : RowVector **quotient** (const RowVector &a, const RowVector &b)

Unary MArray ops.

— : RowVector **operator -** (const RowVector &a)

The `Matrix` classes share the following functions. These will
eventually be part of an `MArray2<T>` class, derived from the
`Array2<T>` class. Then the `Matrix` class will be derived
from the `MArray<T>` class.

Element by element matrix by scalar ops.

— : Matrix **operator +** (const Matrix &a, const double &s)  
— : Matrix **operator -** (const Matrix &a, const double &s)  
— : Matrix **operator \*** (const Matrix &a, const double &s)  
— : Matrix **operator /** (const Matrix &a, const double &s)

Element by element scalar by matrix ops.

— : Matrix **operator +** (const double &s, const Matrix &a)  
— : Matrix **operator -** (const double &s, const Matrix &a)  
— : Matrix **operator \*** (const double &s, const Matrix &a)  
— : Matrix **operator /** (const double &s, const Matrix &a)

Element by element matrix by matrix ops.

— : Matrix **operator +** (const Matrix &a, const Matrix &b)  
— : Matrix **operator -** (const Matrix &a, const Matrix &b)

— : Matrix **product** (const Matrix &a, const Matrix &b)  
— : Matrix **quotient** (const Matrix &a, const Matrix &b)

Unary matrix ops.

— : Matrix **operator -** (const Matrix &a)

The `DiagMatrix` classes share the following functions. These will
eventually be part of an `MDiagArray<T>` class, derived from the
`DiagArray<T>` class. Then the `DiagMatrix` class will be
derived from the `MDiagArray<T>` class.

Element by element MDiagArray by scalar ops.

— : DiagMatrix **operator \*** (const DiagMatrix &a, const double &s)  
— : DiagMatrix **operator /** (const DiagMatrix &a, const double &s)

Element by element scalar by MDiagArray ops.

— : DiagMatrix **operator \*** (const double &s, const DiagMatrix &a)

Element by element MDiagArray by MDiagArray ops.

— : DiagMatrix **operator +** (const DiagMatrix &a, const DiagMatrix &b)  
— : DiagMatrix **operator -** (const DiagMatrix &a, const DiagMatrix &b)

— : DiagMatrix **product** (const DiagMatrix &a, const DiagMatrix &b)

Unary MDiagArray ops.

— : DiagMatrix **operator -** (const DiagMatrix &a)
